# Retrospective cohort study to evaluate the continuous use of anticholesterolemics and diuretics in patients with COVID-19

**DOI:** 10.3389/fmed.2023.1252556

**Published:** 2024-01-11

**Authors:** Fabrício Marcus Silva Oliveira, Mônica Maria Magalhães Caetano, Ana Raquel Viana de Godoy, Larissa Lilian de Oliveira, Juliana Vaz de Melo Mambrini, Marina Santos Rezende, Marina Pinheiro Rocha Fantini, Tiago Antônio De Oliveira Mendes, Nayara Ingrid Medeiros, Henrique Cerqueira Guimarães, Jacqueline Araújo Fiuza, Soraya Torres Gaze

**Affiliations:** ^1^Cellular and Molecular Immunology Group, Rene Rachou Institute, Oswaldo Cruz Foundation, Belo Horizonte, Minas Gerais, Brazil; ^2^Risoleta Tolentino Neves Hospital, Belo Horizonte, Brazil; ^3^ECMO Minas, Belo Horizonte, Minas Gerais, Brazil; ^4^Materdei Hospital, Belo Horizonte, Minas Gerais, Brazil; ^5^Department of Biochemistry and Molecular Biology, Federal University of Viçosa, Viçosa, Minas Gerais, Brazil; ^6^Department of Morphology, Federal University of Minas Gerais, Belo Horizonte, Minas Gerais, Brazil

**Keywords:** coronavirus disease 2019, SARS-CoV-2, biomarkers, antihypercholesterolemic, diuretics

## Abstract

**Purpose:**

The purpose of this study is to evaluate the interference of the continuous use of drug classes in the expression of biomarkers during the first week of hospitalization and in the prognosis of patients with COVID-19.

**Methods:**

The patients diagnosed with COVID-19 and confirmed with SARS-CoV-2 by RT-qPCR assay underwent the collection of fasting whole blood samples for further analysis. Other data also extracted for this study included age, sex, clinical symptoms, related comorbidities, smoking status, and classes of continuous use. Routine serum biochemical parameters, including alanine aminotransferase, aspartate aminotransferase, lactate dehydrogenase, C-reactive protein, N-terminal fragment of B-type natriuretic peptide, and cardiac troponin, were measured.

**Results:**

In this cross-sectional study, a total of 176 patients with COVID-19 hospitalizations were included. Among them, 155 patients were discharged (88.5%), and 21 patients died (12%). Among the drug classes evaluated, we verified that the continuous use of diuretic 4.800 (1.853–11.67) (*p* = 0.0007) and antihypercholesterolemic 3.188 (1.215–7.997) (*p* = 0.0171) drug classes presented a significant relative risk of death as an outcome when compared to the group of patients who were discharged. We evaluated biomarkers in patients who used continuous antihypercholesterolemic and diuretic drug classes in the first week of hospitalization. We observed significant positive correlations between the levels of CRP with cardiac troponin (*r* = 0.714), IL-6 (*r* = 0.600), and IL-10 (*r* = 0.900) in patients who used continuous anticholesterolemic and diuretic drug classes and were deceased. In these patients, we also evaluated the possible correlations between the biomarkers AST, NT-ProBNP, cardiac troponin, IL-6, IL-8, and IL-10. We observed a significantly negative correlations in AST levels with NT-ProBNP (*r* = −0.500), cardiac troponin (*r* = −1.00), IL-6 (*r* = −1.00), and IL-10 (*r* = −1.00) and a positive correlation with IL-8 (*r* = 0.500). We also observed significant negative correlation in the levels of NT-ProBNP with IL-10 (*r* = −0.800) and a positive correlation with cardiac troponin (*r* = 0.800). IL-6 levels exhibited positive correlations with cardiac troponin (*r* = 0.800) and IL-10 (*r* = 0.700).

**Conclusion:**

In this study, we observed that hospitalized COVID-19 patients who continued using anticholesterolemic and diuretic medications showed a higher number of correlations between biomarkers, indicating a poorer clinical prognosis. These correlations suggest an imbalanced immune response to injuries caused by SARS-CoV-2.

## Introduction

Coronavirus disease 2019 (COVID-19) is caused by severe acute respiratory syndrome coronavirus 2 (SARS-CoV-2) (1, 2). Most of its patients have mild to moderate symptoms (81%), including mild fever, fatigue, dry cough, headache, sore throat, myalgia, diarrhea, vomiting, chills, loss of smell, and loss of taste. However, approximately 14% of infected patients progress to pneumonia and may require ventilation in an intensive care unit (ICU), and 5% will eventually develop more critical manifestations such as acute respiratory distress syndrome (ARDS), septic shock, and multiple organ dysfunction or failure, which may progress to death (3–8). In addition to the symptoms developed during the progression of COVID-19, 40% of surviving patients, regardless of whether they were symptomatic or not, have sequelae after the acute phase, called “Post-acute Sequelae of COVID-19 (PASC)” or “long-COVID” with features that include dyspnea, fatigue, chest pain, cognitive decline, and multi-organ damage, especially chronic lung disease (9–13).

Despite the control of the pandemic, due to the availability of vaccines, studies aimed at identifying and understanding factors that alter the clinical status of patients with COVID-19 are still needed. Mutations in the spike (S) protein of SARS-CoV-2 contribute to re-infections, deserving attention from health professionals (14). Health professionals need reliable information related to hospitalized patients with COVID-19 to support the decisions and conduct of the health team regarding care. Biomarkers have been used as important tools in the diagnosis, prognosis, and outcome of COVID-19 (15, 16). Other findings used to guide the health team refer to some comorbidities and pre-existing conditions presented by patients, which are usually followed by a poor prognosis (17, 18). This information, combined with other strategies, such as the development of vaccines and new drugs, contributed to ensuring success in controlling the pandemic caused by SARS-CoV-2 (19–21).

Several groups have studied comorbidities and COVID-19 (17, 18); however, the evaluation of chronic drug use due to those comorbidities during COVID-19 still lacks information. Among the studies documenting this relationship, those known as ACE inhibitors (ACE-Inhs.) and angiotensin 1 receptor (AT1-R) blockers (ARBs) deserve to be highlighted. These drugs have been linked to increasing the expression of angiotensin-converting enzyme 2 (ACE2), the enzyme that SARS-CoV-2 binds to infect host cells. Diuretics are another class of continuous use drugs that have recently been evaluated for their role in the prognosis of COVID-19 (22). Researchers reported that among patients hospitalized with COVID-19, the use of diuretics did not have a significant impact on the mortality or severity of the illness (22). However, data on the role of diuretics in patients with COVID-19 are limited, and some inferences are derived from studies involving patients with acute respiratory distress syndrome (ARDS) (23).

Therefore, given the scarcity of studies that propose to evaluate whether the continued use of diuretics and other classes of drugs such as anticholesterolemics, adrenergic antagonists, non-steroidal anti-inflammatory drugs (NSAIDs), angiotensin receptor blockers (ARBs), ACE inhibitors, calcium channel blockers (CCBs), and psychotropics. We performed a retrospective study with the objective of determining whether classes of continuous use drugs impact the prognosis of hospitalized patients with COVID-19. In addition, we evaluated whether the continued use of these drugs interferes with the correlations of biological biomarkers in COVID-19. This study contributes clinical information about patients with COVID-19 that can guide professionals’ approaches.

## Population, materials, and methods

### Study design and data collection

This observational retrospective study was conducted from 21 July 2020 to 20 March 2021 and included 176 hospitalized patients diagnosed with COVID-19 at MaterDei Hospital or Risoleta Tolentino Neves Hospital, both in Belo Horizonte, Minas Gerais, Brazil. After admission with clinical symptoms of COVID-19, throat swab specimens were obtained for SARS-CoV-2 examination. The specimen suspensions were used for a real-time reverse transcription polymerase chain reaction (RT-qPCR) assay of SARS-CoV-2 RNA, which confirmed COVID-19 in all patients enrolled in this study. The diagnosis and classification of COVID-19 followed the guidelines and management of the Brazil National Health Council for COVID-19 (24). Patients who were hospitalized for other reasons and later diagnosed with COVID-19, pregnant women, and patients who were transferred to another hospital were excluded from the analysis.

It is important to note that during the time of this study, there was no available vaccine approved for use in Brazil. Therefore, the cohort used here is unvaccinated and did not participate in any clinical trials.

**Figure 1:**
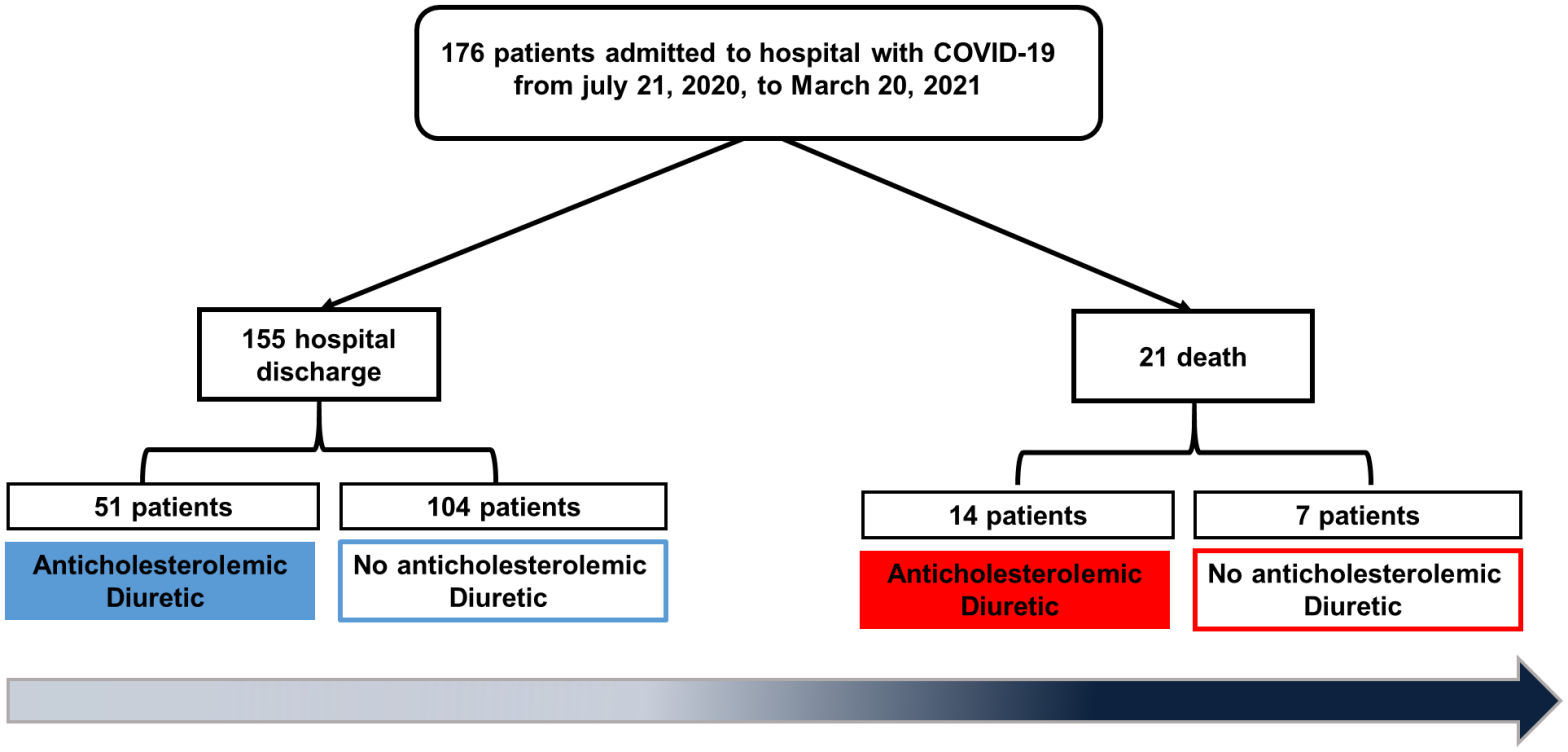
Flow diagram of this study.

The patients diagnosed with COVID-19 and confirmed with SARS-CoV-2 by RT-qPCR assay underwent collection of fasting whole blood samples in tubes treated with EDTA anticoagulant for further analysis within 30 min. Routine peripheral blood cells, including white blood cells, hemoglobin, platelets, lymphocytes, neutrophils, and monocytes, were analyzed by the Beckman Coulter DxH 800 automated blood analyzer and related reagents (Beckman, CA, United States). Routine serum biochemical parameters, including alanine aminotransferase (ALT), aspartate aminotransferase (AST), lactate dehydrogenase, C-reactive protein (CRP), N-terminal fragment of B-type natriuretic peptide (NT-ProBNP), and cardiac troponin, were measured by the Beckman Coulter AU automatic biochemical analyzer and related reagents (Beckman, CA, USA). All tests were performed under hospital sample routine management. This study included biochemical and hematological tests that were performed with samples only from the first week of hospitalization for the patients. Figure 1 shows the experimental design, illustrating screening and inclusion criteria.

Moreover, 10 mL of blood was collected in heparin and centrifuged at 200 g for 10 min at room temperature for plasma separation. Plasma was then frozen in 1 mL aliquot at -80°C and kept until use.

Other data also extracted for this study included age, sex, related comorbidities, smoking status, and drug classes of continuous use. The pharmacological classes, as well as the drugs belonging to these classes evaluated in this study, are described in Table 1. This study was approved by the ethics board at Rene Rachou Institute – Fiocruz MG (CAAE 30399620.0.0000.5091; approval number 3.946.009), and informed consent were obtained from all patients upon hospital admission.

**Table 1:** Medications and drug classes in use by COVID-19 cohort.

Pharmacological class	Drugs (Label name in Brazil)
Adrenergic blocker	Ablock, Atenolol, Carvedilol, Doxazosin, Methyldopa, Metoprolol, Neblock, Nebivolol, Propranolol, Selozok, Bisoprolol
Non-steroidal anti-inflammatory	Dipirona, Ibuprofen, Paracetamol, Resfenol, Somalgin, Acetylsalicylic acid
Angiotensin II receptor antagonists	Atacand, Aradois, Benicar, Diovan, Losartan, Olmesartan, Olmetec
Angiotensin-converting enzyme (ACE) inhibitors	Enalapril, Captopril
Calcium Channel Blocker	Zanidip, Verapamil, Roxflan, Novanlo, Nifedipino, Anlodipino
Anticholesterolemic	Atorvastatin, Ciprofibrato, Ezetimibe, Fenoterol, Rosuvastatin, Rozucor, Sinvastatina, Trezor
Diuretic	Clorana, Clortalidona, Espironolactona, Furosemide, Hidroclorotiazida, Indapen
Hypoglycemic	Forxiga, Galvus met, Glibenclamide, Gliclazide, Glifage, Insulina, Jardiance, Metformin, Xigduo
Psychotropic	Alprazolam, Amitriptyline, Bupropion, Carbamazepine, Clonazepam, Chlorpromazine, Concerta, Desvenlafaxine, Duloxetine, Fluoxetine, Gabapentin, Haloperidol, Lorazepam, Pregabalin, Primidone, Quetiapine, Reconter, Risperidone, Rivotril, Sertraline, Topiramate, Venlafaxine, Zolpidem

According to the outcome, the patients were divided into two groups: the discharged group and the deceased group. The following data were collected: demographic data, clinical symptoms, chronic comorbidities, laboratory results at admission, clinical manifestations, continuous use of medications, and prognostic outcomes. At least two trained staff collected and reviewed data to avoid subjective biases.

### Cytokines measurement

The Human Cytokine Antibody-immobilized Magnetic Bead Panel (Bio-Plex Pro Human Cytokine 27-plex Assay) was used to detect the concentration of interleukin 6 (IL-6), interleukin 8 (IL-8), and interleukin 10 (IL-10) cytokines in the plasma of SARS-CoV-2-infected patients. The tests were carried out according to the manufacturer’s instructions. The samples were analyzed in the Luminex 200 System (Merck).

### Statistical analysis

All statistical analyzes were performed using GraphPad Prism 8 software (GraphPad Inc., United States). Categorical variables were presented as numbers and percentages. When comparing groups with only on equalitative variable, the Kruskal Wallis tests followed by Dunn’s post-test (non-parametric data), the results were presented in terms of median (Minimum-Maximum interquartile range). A chi-square statistical hypothesis test was also used to compare two categorical variables, and the results were presented as relative risk and confidence intervals of 95%. The relationships between variables were correlated using the Spearman rank coefficients. The results were presented as a correlation coefficient r. The tests were considered statistically significant when the value of p was ≤0.05.

## Results

### Demographic parameters and comorbidities

In this cross-sectional study, a total of 176 patients with COVID-19 hospitalizations were included. Among them, 155 patients were discharged (88.5%), and 21 patients died (12%). When we verified the sex distribution of affected patients, male patients were the most affected, making a total of 103 compared to 73 female patients (Table 2). However, in this study, sex was not an important factor in the condition of death as an outcome. We observed that advanced age was a significant risk factor for death. The average age of deceased patients was significantly higher (69.09 ± 15.36) years old in relation to patients discharged (58.47 ± 14.24) years old (*p* = 0.0018), when this primary outcome was considered (Table 2).

**Table 2:** Demographic parameters and associated comorbidities.

Parameters		Overall *n* = 176	Hospital discharge *n* = 155	Deaths *n* = 21	*p*-value	Relative Risk (CI 95%)
AGE years Mean ± SD	Female	59.74 (±14.74)	58.47 (±14.24)	69.09 (±15.36)	0.0018	–
	Male	62.68 (±14.18)	60.83 (±14.04)	74.1 (±9.036)	0.0052	–
		57.70 (±14.85)	56.88 (±14.22)	64.55 (±18.72)	0.1059	–
SEX (*n*.%)	Female	73	63 (86.30)	10 (13.70)	0.5427	1.283 (0.5840–2.796)
	Male	103	92 (89.32)	11 (10.67)	0.5427	0.7796 (0.3577–1.712)
Comorbidity (*n*.)						
Obesity		32	27	5	0.4762	1.406 (0.5566–3.320)
Diabetes		42	34	8	0.1030	1.963 (0.8758–4.249)
Hypertension		92	77	15	0.0611	2.283 (0.9648–5.502)
Chronic Cardiovascular Disease		28	21	7	*0.0200	2.643 (1.160–5.657)

Data are presented as mean ± standard deviation (SD) or number (percentage) or are presented as relative risk and confidence intervals (CIs) 95%. A two-tailed *p*-value of ≤ 0.05 was considered significant. Bold values and (*) show significant diferences.

The comorbidities, signs and symptoms, and COVID-19 clinical manifestations were compared between deceased and discharged patients (Table 2; Supplementary File S1). Obesity, diabetes, hypertension, and chronic cardiovascular diseases were evaluated separately in our study. Among those conditions, only chronic cardiovascular disease [2.643 (1.160–5.657)] (*p* = 0.0200) presented a significant relative risk for patients with deceased outcomes (Table 2). In our study, upon hospital admission, the oxygen saturation in ambient air, typical signs and symptoms of COVID-19, as well as other clinical manifestation parameters, were evaluated. However, none presented a significant relative risk for the outcome of death. The characterization of signs, symptoms, and clinical manifestations is shown in Supplementary File S1.

### The use of diuretics and anticholesterolemics by patients with COVID-19 related to a poor prognosis

Due to the high worldwide prevalence of morbidities such as obesity, diabetes, hypertension, and chronic cardiovascular disease, an increasing number of people are taking continuous drugs to treat those comorbidities. It is expected that with the right medication and guidance, the patients can achieve a near-normal life. However, we hypothesized that the continued use of drug classes could be related to the outcome of patients hospitalized with COVID-19. In order to obtain an answer, at the time of admission, we collected information on which classes of drugs were used continuously by the cohort and verified whether there was a relative risk for death (Table 1). For the analyzes involving the class of drugs of continuous use, we excluded from the analysis patients with chronic cardiovascular disease because they already present a significant relative risk for death, as verified in this study.

Among the drug classes evaluated, we verified that the continuous use of diuretic 4.800 (1.853–11.67) (*p* = 0.0007) and antihypercholesterolemic 3.188 (1.215–7.997) (*p* = 0.0171) drug classes presented a significant relative risk of death as an outcome when compared to the group of patients who were discharged (Table 3). Moreover, the continuous use of other drug classes, such as adrenergic antagonists, non-steroidal anti-inflammatory drugs (NSAIDs), angiotensin receptor blockers (ARBs), ACE inhibitors, calcium channel blockers (CCBs), and psychotropics, was also evaluated and showed no risk of death in this cohort (Table 3). Thereby, we demonstrate that patients in diuretic and anticholesterolemic classes may have a higher risk of death when hospitalized due to COVID-19.

**Table 3:** Drugs classes used continuously by patients hospitalized with COVID-19.

Drug class	Overall *n* = 147	Hospital discares *n* = 133	Deaths *n* = 14	*p*-value	Relative Risk (CI 95%)
Adrenergic blocker	18	16	2	0.6858	1.176 (0.3024–4.015)
Non-steroidal anti-inflammatory	19	15	4	0.0644	2.716 (0.9451–7.092)
Angiotensin II receptor antagonists	41	36	5	0.5354	1.436 (0.5242–3.799)
ACE inhibitors	15	13	1	0.6373	1.467 (0.3783–4.875)
Calcium channel blocker	22	18	4	0.1261	2.309 (0.7998–6.134)
Anticholesterolemic	28	22	6	*0.0171	3.188 (1.215–7.997)
Diuretic	20	14	6	*0.0007	4.800 (1.853–11.67)
Hypoglycemic	30	26	4	0.4853	1.560 (0.5367–4.267)
Psychotropics	18	15	3	0.3814	1.955 (0.6098–5.581)

Data are presented as relative risk and confidence intervals (CIs) 95%.

A two-tailed *p*-value of ≤ 0.05 was considered significant. Bold values and (*) show significant diferences.

### Molecular biomarkers of blood levels in the first week of hospitalization appear to predict the COVID-19 outcome

The determination of biochemical and inflammatory biomarkers has been shown to be an important tool in the diagnosis, prognosis, and outcome of COVID-19. In our study, biochemical and inflammatory biomarkers were evaluated in the first week of patients hospitalization (Table 3). In our study, deceased patients had elevated levels of AST 59 (27–173) (*p* = 0.0231), NT-ProBNP 337.0 (67.40–1,010) (*p* = 0.0041), and cardiac troponin 15.07 (2.180–34.20) (*p* = 0.0037) in the first week of hospitalization, when compared with discharged patients who had AST 47.42 (16.50–98.09), NT-ProBNP 131.5 (0–488), and cardiac troponin 6.200 (1.500–21.36) levels. In addition, patients in the deceased group also had high levels of CRP 163.7 (33.10–360.1) (*p* = 0.0398), IL-6 7.66 (0.87–37.21) (*p* = 0.0135), IL-8 3.71 (2.65–8) (*p* = 0.044), and IL-10 8.53 (2.1–13.52) (*p* = 0.0097) when compared with discharged patients who exhibited CRP 86.90 (2.29–298.3), IL-6 4.95 (0.03–15.99), IL-8 2.50 (0.38–7.52), and IL-10 2.97 (0.33–13.38) (Table 4). In order to verify if there were differences in the blood cell counts and related markers of discharged or deceased patients, we evaluated red cells, hemoglobin, hematocrit, platelets, leucocyte count, segmented neutrophil, lymphocyte count, and neutrophil/lymphocyte ratio (NLR) in peripheral blood samples from patients with COVID-19 in the first week of hospitalization. No differences were observed between the blood counts of the discharge or death groups in this cohort (Supplementary File S2).

**Table 4:** Biomarkers observed in patients with COVID-19 in the first week of hospitalization.

Parameters	Overall *n* = 176	Hospital discharge *n* = 155	Deaths *n* = 21	*p*-value	Relative risk (CI 95%)
Clinical (*n*.)					
Oxygen saturation Mean ± SD	90.90 (±3.549)	91.11 (±3.434)	88.40 (±4.083)	0.0933	–
Fever	114	101	13	0.7694	0.8838 (0.4000–1.994)
Dry cough	116	101	15	0.5696	1.293 (0.5529–3.118)
Cough with secretion	31	26	5	0.5399	1.462 (0.5790–3.440)
Coryza	48	40	8	0.2354	1.641 (0.7299–3.580)
Shortness of breathe	77	69	8	0.5778	0.7912 (0.3506–1.761)
Weakness	132	116	16	0.8932	1.067 (0.4408–2.711)
Smell disorders	49	43	6	0.9366	1.037 (0.4308–2.400)
Taste disorder	25	22	3	0.9999	0.9537 (0.3101–2.642)
Sore throat	27	23	4	0.5359	1.298 (0.4752–3.250)
Diarrhea	55	50	5	0.6165	0.6875 (0.2698–1.686)
Nausea and vomiting	51	42	9	0.1352	1.838 (0.8317–3.970)

Data are presented as median (minimum–maximum and interquartile range). A two-tailed *p*-value of ≤ 0.05 was considered significant.

### Correlations of COVID-19 biomarkers in patients with continuous use of anticholesterolemic and diuretic drug classes

In order to verify the profile of correlations between biochemical and inflammatory biomarkers and whether the continuous use of these drugs could alter the relationship of these biomarkers, we evaluated biomarkers in patients who used continuous antihypercholesterolemic and diuretic drug classes at the first week of hospitalization.

**Figure 2:**
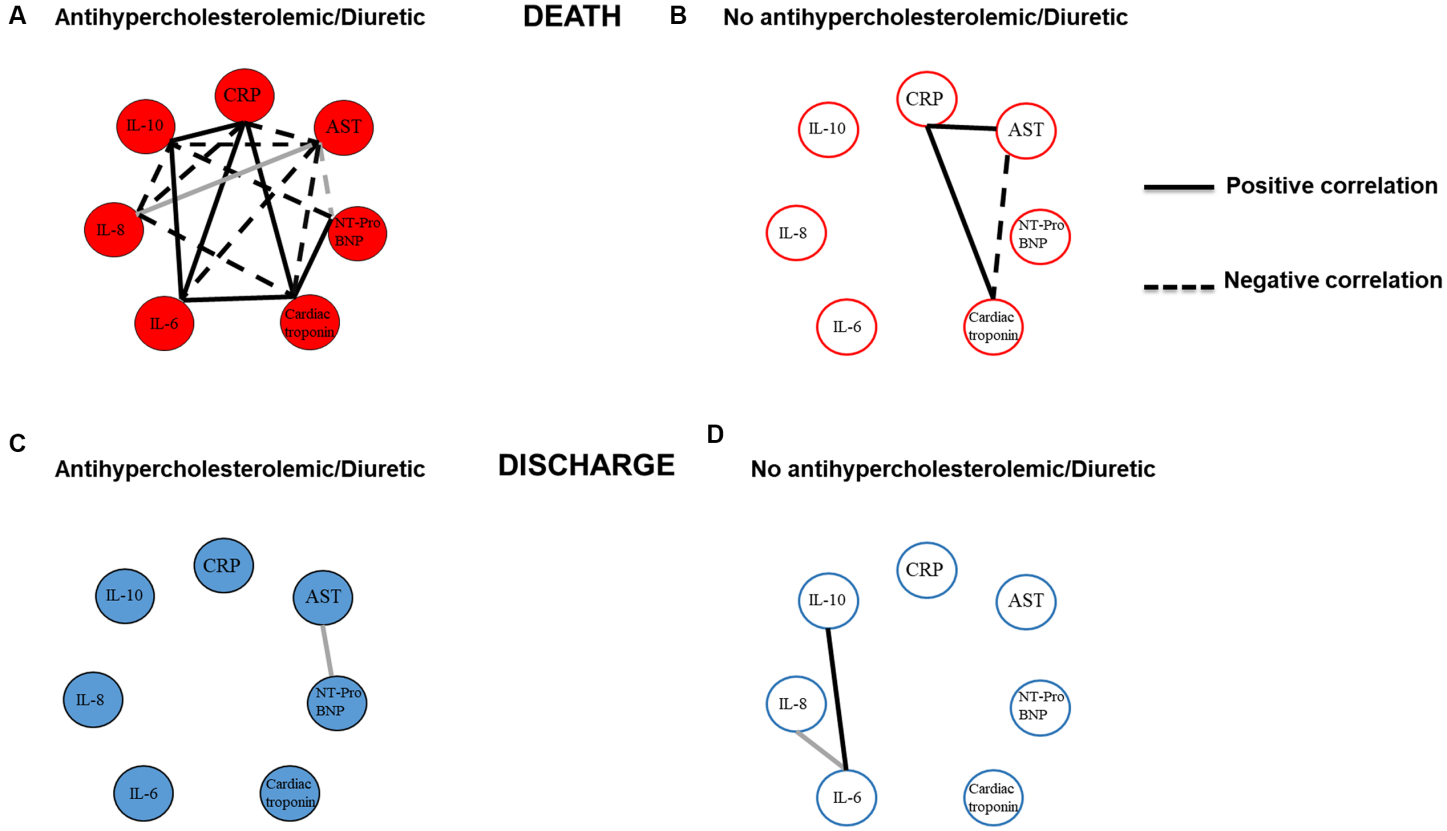
Profile of correlations of COVID-19 biomarkers in patients with continuous use of anticholesterolemics and diuretics. **(A)** Patients who used drugs belonging to the anticholesterolemic and diuretic classes and who died. **(B)** Patients who did not use drugs belonging to the anticholesterolemic and diuretic classes and who died. **(C)** Patients who used drugs belonging to the anticholesterolemic and diuretic classes and who were discharged. **(D)** Patients who were not using drugs belonging to the anticholesterolemic and diuretic classes and who were discharged. Black lines show strong correlation (0.6 to 1.0 or −0.6 to −1.0) and gray lines show moderate correlation (0.5 to 0.59 or −0.5 to −0.59).

We observed significant positive correlations between the levels of CRP with cardiac troponin (r = 0.714), IL-6 (r = 0.600), and IL-10 (r = 0.900) in patients who used continuous anticholesterolemic and diuretic drug classes and were deceased (Figure 2A and Supplementary File S3A). On the other hand, when verifying the correlation of CRP with AST (r = −0.700) and IL-8 (r = −0.600), we observed significant negative correlations between these biomarkers (Figure 2A and Supplementary File S3A). In these patients, we also evaluated the possible correlations between the biomarkers AST, NT-ProBNP, cardiac troponin, IL-6, IL-8, and IL-10.

We observed significant negative correlations in AST levels with NT-ProBNP (*r* = −0.500), cardiac troponin (*r* = −1.00), IL-6 (*r* = −1.00), and IL-10 (*r* = −1.00) and a positive correlation with IL-8 (*r* = 0.500) (Figure 2A and Supplementary File S3A). We also a observed significant negative correlation in the levels of NT-ProBNP with IL-10 (*r* = −0.800) and a positive correlation with cardiac troponin (*r* = 0.8000). IL-6 levels exhibited positive correlations with cardiac troponin (*r* = 0.800) and IL-10 (*r* = 0.7) (Figure 2A and Supplementary File S3A).

When verifying the possible correlations of biomarkers in deceased patients who are not using continuous anticholesterolemic and diuretic drug classes, we observed a significant positive correlation of AST with CRP (*r* = 0.900), positive correlation of CRP with cardiac troponin (*r* = 0.900), and a negative correlation of AST with cardiac troponin (*r* = −0.600) (Figure 2B and Supplementary File S3B).

The analysis of possible correlations between the biomarkers in discharged patients who used continuous antihypercholesterolemic and diuretic drugs showed a different profile of correlations. We observed that the number of correlations between biomarkers in these patients was reduced compared to patients who used continuous anticholesterolemic and diuretic drug classes and who had death as the outcome. It was only observed that AST levels showed a positive correlation with NT-ProBNP (*r* = 0.590) (Figure 2C and Supplementary File S3C). Regarding discharged patients who did not use these drugs, it was observed that Il-6 levels showed positive correlations with IL-8 (*r* = 0.510) and IL-10 (*r* = 0.600) (Figure 2D and Supplementary File S3D).

Collectively, these results demonstrate that patients using anticholesterolemic and diuretic drug classes and who had death as the outcome showed the highest number of correlations among the biomarkers. In addition, the results also demonstrate that among the biomarkers evaluated, CRP and AST had the highest number of significant correlations, demonstrating the need to direct attention to this biomarker during the first week of hospitalization for COVID-19.

## Discussion

The success of controlling the pandemic caused by SARS-CoV-2 was largely due to the development of vaccines and new drugs and the identification of biological biomarkers of progression to severe and fatal forms of the disease (15, 19–21, 25). In addition, it is important that reliable information related to clinical manifestations and biological parameters related to COVID-19 is available from the health team to support decisions and guide conduct regarding the care provided to hospitalized patients. In this study, we identified that the continuous use of classes of antihypercholesterolemic drugs and diuretics by patients with COVID-19 is associated with interference in the expression of biomarkers in the first week of hospitalization and with a significant risk of death as an outcome in these patients. Moreover, we verified whether the demographic data, existing comorbidities, and clinical and laboratory parameters identified during the first week of hospitalization of patients had any relationship with the final outcome.

COVID-19 is a heterogeneous infectious disease, and the understanding of host factors contributes to determining disease progression and outcome. The main risk factors for severe manifestations of COVID-19 include advancing age, male gender, obesity, smoking, and chronic comorbidity conditions such as hypertension, type 2 diabetes mellitus, and others (3–5, 18, 26–28). We accessed data obtained during the first week of COVID-19 patients’ hospitalization during the period from 21 July 2020 to 20 March 2021. We also verified the relationship of these data with the patients’ outcomes, divided into discharged or deceased. In corroboration with other studies, which demonstrate that older age is a risk factor for severe COVID-19 (4, 5, 18, 28), we also observed that deceased patients were indeed older. Interestingly, in relation to the male gender as a risk factor reported in the literature (29–32), the same was not observed by us since female patients in this group had a higher mean age than male patients. Therefore, the male sex of the cohort included in our study was not a risk factor for the outcome of death. We emphasize that female patients in the deceased cohort were, on average, 10 years older than male patients. This observation suggests that the difference between our results and those of other studies could be due to the advanced age of female patients, which allows us to infer that advanced age is a more important risk factor than sex.

The classic symptoms of COVID-19 have already been characterized and include fever, dry cough, fatigue, shortness of breath, chills, muscle pain, headache, gastric disturbances, and weight loss (6, 33). Although we had observed symptoms like those already described in the literature, in the present study, no symptoms presented during the first week of hospitalization were considered a significant risk for the deceased outcome.

Other studies previously described the importance of hemogram tests in providing critical COVID-19 prognostic markers and benefiting healthcare staff in assisting the triage and care of infected patients (34–39). In this study, in our cohort, there were no significant changes and no relationship between those counts and the outcome of COVID-19 patients. This observation reinforces that COVID-19 has many levels of affecting people, and studies with different populations and disease backgrounds should be performed in different countries.

Considering clinical and epidemiological data on COVID-19, it is suggested that comorbidities and pre-existing conditions, such as cardiovascular disease, chronic kidney disease, smoking habits, chronic lung diseases (particularly COPD), diabetes mellitus, hypertension, rheumatoid arthritis, immunosuppression, and obesity, predispose patients to an unfavorable prognosis and consequently an increased risk of death (1, 5, 27, 40–42). The current study found a similar result to those previously reported, confirming that. In our evaluated cohort, chronic cardiovascular disease was associated with a deceased outcome in COVID-19 patients. Indeed, previous studies have documented that COVID-19 patients have the potential to develop severe heart failure, either due to deteriorating pre-existing cardiac dysfunction or due to newly developed cardiomyopathy and myocarditis, which are indicative of a poor prognosis (4, 18).

In addition, we verified whether the continuous use of drugs to treat previous comorbidities could be related to the poor prognosis of COVID-19. In this study, we identified that the continuous use of diuretics and anticholesterolemics was related to the deceased outcome in the evaluated cohort. Currently, information on the impact of continued use of these classes of drugs on COVID-19 is scarce.

Studies demonstrating hyponatremia (the most common electrolytic disorder observed in clinical practice) was associated with an increased risk of death in COVID-19 patients (43, 44). We hypothesized that the relationship between the continued use of diuretics and the observed deceased outcome observed in our study could be due to a possible clinical manifestation of hyponatremia as a side effect or aggravation of the continued use of this class of drugs (45, 46). Indeed, hyponatremia is the most common electrolyte disturbance observed in clinical practice and is associated with the outcome of death (43). Merino et al. (47) reported hemodynamic disorders or the immune response present in COVID-19, which can damage kidney cells, causing electrolyte disturbances. In fact, kidney cells express angiotensin-converting enzyme 2 and receptors used by SARS-CoV-2 as gateways; subsequently, viral replication occurs in infected cells, followed by cell injury (47, 48). Another pathophysiological mechanism that may explain renal impairment in COVID-19 is induced by inflammatory cytokines. The literature reports that the cytokine cascade can cause a series of renal alterations, such as acute kidney injury, tubular necrosis, renal proximal tubule dysfunction, and glomerulopathy, which are most often accompanied by electrolyte abnormalities (48–50).

Unlike what was observed in our study, Guragai et al. (22) reported that, despite having higher levels of biomarkers, patients hospitalized with COVID-19 who used diuretics did not have a significant impact on mortality or disease severity. However, it is worth noting that only two classes of diuretics, furosemide and hydrochlorothiazide, were evaluated, while in our study, patients used the following drugs: chlorine, chlortalidone, spironolactone, furosemide, hydrochlorothiazide, and indapen. In addition, the distinction of patients into severe and non-severe COVID-19 was carried out, which was not carried out in our study, which could justify the divergence of the two studies. Given the above and supported by our results, we suggest that the continuous use of diuretics may favor the appearance or worsen the clinical picture of hyponatremia in patients with COVID-19. Therefore, we strongly recommend COVID-19 hospitalized patients have their blood ion balance monitored.

Regarding the anticholesterolemic drugs, we believe that by reducing cholesterol levels, they contribute to the severity of COVID-19. Several studies report that reduced levels of total cholesterol, low-density lipoprotein cholesterol (LDL-C), and high-density lipoprotein cholesterol (HDL-C) are associated with severity and mortality in COVID-19 patients (51–56). A possible explanation for this relationship would be the ability of HDL-C to bind and neutralize viruses and toxic bacterial substances, such as lipopolysaccharides (LPS) (57–59) and lipoteichoic acid (60). Moreover, HDL-C can block the penetration of certain viruses into cells, reducing tissue invasion (61). Further studies are needed to elucidate this relationship. On the other hand, patients presenting a clinical picture of hyperlipidemia also deserve attention, as they are at high risk of developing severe COVID-19 and death (62–65). Researchers suggest that these patients are prone to developing complications due to the combination of underlying chronic systemic endothelial dysfunction caused by lifelong hypercholesterolemia associated with acute direct endothelial attacks by both the virus and the excessive host immune-inflammatory response (23, 24, 65–67). The complexity around cholesterol levels in patients with COVID-19 needs more reinforcement and attention for hospitalized patients, as instructed in conduct guides developed by other authors (63, 64). In this study, we suggest the need for further studies addressing the relationship between diuretics and anticholesterolemics in patients with COVID-19 in order to understand the mechanism by which these drugs impact the pathophysiology of this disease, leading to a poor prognosis.

The determination of biomarkers has been an important tool for diagnosis, prognosis, and injury in the outcome of damaged pathogenic mechanisms as well as organs (7, 68–70). We evaluated biochemical and inflammatory biomarkers in the cohort involved in this study and related them to the outcome of COVID-19. The high levels of some biomarkers are related to a poor prognosis for COVID-19. Corroborating with other studies (8, 15, 71, 72), we found that deceased patients exhibited elevated levels of NT-ProBNP, AST, cardiac troponin, CRP, IL-6, IL-8, and IL-10 in the first week of hospitalization. The medical team’s choices for the care of COVID-19 patients are mainly guided by laboratory tests, vital signs, and a basic radiological assessment. Our data reinforce the potential of using biomarkers early in COVID-19 patients as potential tools for prognostic indicators.

Previously in this study, we demonstrated that patients in continuous use of drugs belonging to the anticholesterolemic and diuretic classes had the worst outcomes. Furthermore, our group has questioned whether the use of drugs to treat pre-existing conditions by COVID-19 patients could alter the expression of biomarkers. In this view, we aimed to verify whether the continuous use of these drug classes by non-cardiac patients impacted the correlations between biochemical and inflammation biomarkers and what the relationship was with the outcomes. We observed that patients who used these drugs continuously and died presented the highest number of significant correlations when compared to discharged patients. In addition, they also highlighted the highest number of negative correlations, suggesting an important imbalance in the control of the clinical manifestations of COVID-19. As those differences were noted, it is important to mention that only deceased COVID-19 patients in use with both drug classes showed a positive correlation between the important inflammatory cytokine IL-16, CRP, and cardiac troponin. Although CRP showed a negative correlation with the regulatory cytokine IL-10, this could be a circumstantial attempt at regulating systemic inflammation. Moreover, AST showed a negative correlation with IL-10, showing that liver inflammation could be exacerbated. Again, this shows the complexity of COVID-19 manifestations. Therefore, considering the AST correlation results, it could be beneficial to evaluate COVID-19 patients for liver steatosis.

Notably, continuous investment in research is necessary with the aim of deepening knowledge about biomarkers, especially in relation to their role in pathophysiology (73) and correlations with other biological parameters and preconditions presented by COVID-19 patients. There is consensus among the scientific community and health professionals that a fundamental step in the diagnosis and prognosis of COVID-19 is the examination of routine laboratory biomarkers (74, 75), and it is necessary to investigate possible elements that contribute to altering the expression of these biomarkers. In this study, we contributed to identifying the gap to be investigated in future in relation to the imbalance that drugs can cause in biomarkers.

This study has some limitations. First, we have a limitation when comparing the impact of using anticholesterolemics and diuretics on deceased patients. Patients who did not use medication and died were a small number in the cohort. Some patients did not have all the parameters evaluated. The reasons were due to the failure of the health team or problems with hospital logistics. The results described here, especially in relation to drugs for continuous use, cannot be generalized to all patients with COVID-19 since this retrospective study only evaluated hospitalized patients. This study only evaluated the previous use of diuretics and anticholesterolemics on admission, and during the first week of hospitalization, it did not evaluate the impact of using these drugs for the first time in hospitalized patients or the addition of new diuretics. In addition, the clinical parameters reported here refer only to the first week of hospitalization and are associated with the outcome. We did not evaluate the parameters after the first week of hospitalization.

Pre-existing conditions and comorbidities are common observations in the anamnesis performed at the time of admission of hospitalized COVID-19 patients. We know that pre-existing conditions and comorbidities are often accompanied by continuous drug and medication use. We have verified for the first time that hospitalized COVID-19 patients on continuous use of anticholesterolemic and diuretic classes had affected biomarker expression and had experienced a worse prognosis. Patients using anticholesterolemic and diuretic medications display correlations that indicate an imbalance in the immune response, potentially exacerbating the consequential injuries caused by the SARS-CoV-2 infection. Moreover, we found that the CRP and AST biomarkers showed the highest numbers of significant correlations, demonstrating the need to direct attention on these biomarkers during the first week of COVID-19 hospitalization. Finally, we reinforce that the drug classes’ medications and its continuous use need more attention and studies because they could impact the care and prognosis in hospitalized COVID-19 patients.

## Data availability statement

The original contributions presented in the study are included in the article/Supplementary material, further inquiries can be directed to the corresponding author.

## Ethics statement

The studies involving humans were approved by this study received approval from the ethics board at the Rene Rachou Institute – Fiocruz MG (CAAE 30399620.0.0000.5091; approval number 3.946.009). The studies were conducted in accordance with the local legislation and institutional requirements. The participants provided their written informed consent to participate in this study.

## Author contributions

SG, NM, and JF were responsible for grant writing. MC, FO, AG, and LO performed data organization and verification. FO and MC performed data analyzes. FO, SG, and JF drafted the manuscript. MR, MF, and HG were responsible for patients’ care and sample collection. HG, TM, and SG were responsible for the design of the study. JM and TM were responsible for statistical analysis. All authors contributed to the article and approved the submitted version.

## References

[1] Zhu Z Cai T Fan L Lou K Hua X Huang Z. Clinical value of immune-inflammatory parameters to assess the severity of coronavirus disease 2019. Int J Infect Dis. (2020) 95:332–9. doi: 10.1016/j.ijid.2020.04.041, PMID: 32334118 PMC7195003

[2] Whitworth J. COVID-19: a fast evolving pandemic. Trans R Soc Trop Med Hyg. (2020) 114:241–8. doi: 10.1093/trstmh/traa025, PMID: 32198918 PMC7184420

[3] Guan W Ni Z Hu Y Liang W Ou C He J. Clinical characteristics of coronavirus disease 2019 in China. N Engl J Med. (2020) 382:1708–20. doi: 10.1056/NEJMoa2002032, PMID: 32109013 PMC7092819

[4] Chen N Zhou M Dong X Qu J Gong F Han Y. Epidemiological and clinical characteristics of 99 cases of 2019 novel coronavirus pneumonia in Wuhan, China: a descriptive study. Lancet. (2020) 395:507–13. doi: 10.1016/S0140-6736(20)30211-7, PMID: 32007143 PMC7135076

[5] Huang C Wang Y Li X Ren L Zhao J Hu Y. Clinical features of patients infected with 2019 novel coronavirus in Wuhan, China. Lancet. (2020) 395:497–506. doi: 10.1016/S0140-6736(20)30183-5, PMID: 31986264 PMC7159299

[6] Gallo Marin B Aghagoli G Lavine K Yang L Siff EJ Chiang SS. Predictors of COVID-19 severity: a literature review. Rev Med Virol. (2021) 31:1–10. doi: 10.1002/rmv.2146, PMID: 32845042 PMC7855377

[7] Cheng Y Luo R Wang K Zhang M Wang Z Dong L. Kidney disease is associated with in-hospital death of patients with COVID-19. Kidney Int. (2020) 97:829–38. doi: 10.1016/j.kint.2020.03.005, PMID: 32247631 PMC7110296

[8] Wang D Hu B Hu C Zhu F Liu X Zhang J. Clinical characteristics of 138 hospitalized patients with 2019 novel coronavirus-infected pneumonia in Wuhan, China. JAMA. (2020) 323:1061–9. doi: 10.1001/jama.2020.1585, PMID: 32031570 PMC7042881

[9] Schneider EC. Persistent symptoms in patients after acute COVID-19. N Engl J Med. (2020) 383:299–302. doi: 10.1056/NEJMp2014836, PMID: 32412704

[10] Tenforde MW Kim SS Lindsell CJ Billig Rose E Shapiro NI Files DC. Symptom duration and risk factors for delayed return to usual health among outpatients with COVID-19 in a multistate health care systems network — united. MMWR Morb Mortal Wkly Rep. (2020) 69:993–8. doi: 10.15585/mmwr.mm6930e1, PMID: 32730238 PMC7392393

[11] Huang C Huang L Wang Y Li X Ren L Gu X. 6-month consequences of COVID-19 in patients discharged from hospital: a cohort study. Lancet. (2021) 397:220–32. doi: 10.1016/S0140-6736(20)32656-8 33428867 PMC7833295

[12] Fadista J Kraven LM Karjalainen J Andrews SJ Geller F Baillie JK. Shared genetic etiology between idiopathic pulmonary fibrosis and COVID-19 severity. EBioMedicine. (2021) 65:103277. doi: 10.1016/j.ebiom.2021.103277 33714028 PMC7946355

[13] Montani D Savale L Noel N Meyrignac O Colle R Gasnier M. Post-acute COVID-19 syndrome. Eur Respir Rev. (2022) 31:210185–15. doi: 10.1183/16000617.0185-2021 35264409 PMC8924706

[14] Li F. Structure, function, and evolution of coronavirus spike proteins. Annu Rev Virol. (2016) 3:237–61. doi: 10.1146/annurev-virology-110615-042301 27578435 PMC5457962

[15] Ponti G Maccaferri M Ruini C Tomasi A Ozben T. Biomarkers associated with COVID-19 disease progression. Crit Rev Clin Lab Sci. (2020) 57:389–99. doi: 10.1080/10408363.2020.1770685, PMID: 32503382 PMC7284147

[16] Jawad S Shahzad H Naifar N Grey I Arora T Thomas J. Biomarkers of endothelial dysfunction and outcomes in coronavirus disease 2019 (COVID-19) patients: a systematic review and meta-analysis. Psychiatry Res. (2020) 14:293. doi: 10.1016/j.mvr.2021.104224

[17] Ejaz H Alsrhani A Zafar A Javed H Junaid K Abdalla AE. COVID-19 and comorbidities: deleterious impact on infected patients. J Infect Public Health. (2020) 13:1833–9. doi: 10.1016/j.jiph.2020.07.014, PMID: 32788073 PMC7402107

[18] Zhou F. Clinical course and risk factors for mortality of adult in patients with COVID-19 in Wuhan, China: a retrospective cohort study. Med Internet Res. (2020) 3:01–2. doi: 10.24966/MSR-5657/100015 PMC7270627 32171076

[19] Soleimanpour S Yaghoubi A. COVID-19 vaccine: where are we now and where should we go? Expert Rev Vaccines. (2021) 20:23–44. doi: 10.1080/14760584.2021.1875824, PMID: 33435774 PMC7898300

[20] Asselah T Durantel D Pasmant E Lau G Schinazi RF. COVID-19: discovery, diagnostics and drug development [internet]. J Hepatol. (2021) 74:168–84. doi: 10.1016/j.jhep.2020.09.031, PMID: 33038433 PMC7543767

[21] Owji H Negahdaripour M Hajighahramani N. Immunotherapeutic approaches to curtail COVID-19. Int Immunopharmacol. (2020) 88:106924. doi: 10.1016/j.intimp.2020.106924 32877828 PMC7441891

[22] Guragai N Vasudev R Hosein K Habib H Patel B Kaur P. Does baseline diuretics use affect prognosis in patients with COVID-19? Cureus. (2021) 13:e15573. doi: 10.7759/cureus.15573 34277195 PMC8272599

[23] Heart TN. Comparison of two fluid-management strategies in acute lung injury. N Engl J Med. (2006) 354:2564–75. doi: 10.1056/NEJMoa062200 16714767

[24] da Brasil MS. Diagnóstico e Tratamento da COVID-19 TRATAMENTO. Secretaria De Ciência, Tecnologia, Inovação E Insumos Estratégicos Em Saúde. (2020);1:1–398. São Paulo

[25] Bivona G Agnello L Ciaccio M. Biomarkers for prognosis and treatment response in covid-19 patients. Ann Lab Med. (2021) 41:540–8. doi: 10.3343/alm.2021.41.6.540, PMID: 34108281 PMC8203437

[26] Zhu N Zhang D Wang W Li X Yang B Song J. A novel coronavirus from patients with pneumonia in China, 2019. N Engl J Med. (2020) 382:727–33. doi: 10.1056/NEJMoa2001017, PMID: 31978945 PMC7092803

[27] Tajbakhsh A Gheibi Hayat SM Taghizadeh H Akbari A inabadi M Savardashtaki A. COVID-19 and cardiac injury: clinical manifestations, biomarkers, mechanisms, diagnosis, treatment, and follow up. Expert Rev Anti-Infect Ther. (2021) 19:345–57. doi: 10.1080/14787210.2020.1822737, PMID: 32921216

[28] Wu Z McGoogan JM. Characteristics of and important lessons from the coronavirus disease 2019 (COVID-19) outbreak in China. JAMA. (2020) 323:1239–42. doi: 10.1001/jama.2020.2648, PMID: 32091533

[29] Zhang J-J Cao Y-Y Tan G Dong X Wang B-C Lin J. Clinical, radiological, and laboratory characteristics and risk factors for severity and mortality of 289 hospitalized COVID-19 patients. Allergy. (2021) 76:533–50. doi: 10.1111/all.14496, PMID: 32662525 PMC7404752

[30] Wolff D Nee S Hickey NS Marschollek M. Risk factors for Covid-19 severity and fatality: a structured literature review. Infection. (2021) 49:15–28. doi: 10.1007/s15010-020-01509-1, PMID: 32860214 PMC7453858

[31] Zhang J Wang X Jia X Li J Hu K Chen G. Risk factors for disease severity, unimprovement, and mortality in COVID-19 patients in Wuhan, China. Clin Microbiol Infect. (2020) 26:767–72. doi: 10.1016/j.cmi.2020.04.012, PMID: 32304745 PMC7159868

[32] Ebinger JE Achamallah N Ji H Claggett BL Sun N Botting P. Pre-existing traits associated with Covid-19 illness severity. PloS one. (2020) 15:e0236240. doi: 10.1371/journal.pone.0236240, PMID: 32702044 PMC7377468

[33] Gao Z Xu Y Sun C Wang X Guo Y Qiu S. A systematic review of asymptomatic infections with COVID-19. J Microbiol Immunol Infect. (2021) 54:12–6. doi: 10.1016/j.jmii.2020.05.001, PMID: 32425996 PMC7227597

[34] Zhang L Huang B Xia H Fan H Zhu M Zhu L. Retrospective analysis of clinical features in 134 coronavirus disease 2019 cases. Epidemiol Infect. (2020) 148:e199. doi: 10.1017/S0950268820002538, PMID: 32878654 PMC7487751

[35] Palladino M. Complete blood count alterations in COVID-19 patients. Biochem Med. (2021) 31:403–15. doi: 10.11613/BM.2021.030501, PMID: 34658642 PMC8495616

[36] Lippi G Plebani M Henry BM. Thrombocytopenia is associated with severe coronavirus disease 2019 (COVID-19) infections: a meta-analysis. Clin Chim Acta. (2020) 506:145–8. doi: 10.1016/j.cca.2020.03.022, PMID: 32178975 PMC7102663

[37] Qin C Zhou L Hu Z Zhang S Yang S Tao Y. Dysregulation of immune response in patients with COVID-19 in Wuhan, China. Clin Infect Dis. (2020) 71:762–8. doi: 10.2139/ssrn.3541136 32161940 PMC7108125

[38] Yamada T Wakabayashi M Yamaji T Chopra N Mikami T Miyashita H. Value of leukocytosis and elevated C-reactive protein in predicting severe coronavirus 2019 (COVID-19): a systematic review and meta-analysis. Clin Chim Acta. (2020) 509:235–43. doi: 10.1016/j.cca.2020.06.008, PMID: 32533986 PMC7832771

[39] Zhang B Zhou X Zhu C Song Y Feng F Qiu Y. Immune phenotyping based on the neutrophil-to-lymphocyte ratio and IgG level predicts disease severity and outcome for patients with COVID-19. Front Mol Biosci. (2020) 7:1–7. doi: 10.3389/fmolb.2020.00157 32719810 PMC7350507

[40] Cecconi M Piovani D Brunetta E Aghemo A Greco M Ciccarelli M. Early predictors of clinical deterioration in a cohort of 239 patients hospitalized for Covid-19 infection in Lombardy, Italy. J Clin Med. (2020) 9:1–16. doi: 10.3390/jcm9051548 PMC7290833 32443899

[41] Guo W Li M Dong Y Zhou H Zhang Z Tian C. Diabetes is a risk factor for the progression and prognosis of COVID-19. Diabetes Metab Res Rev. (2020) 36:1–9. doi: 10.1002/dmrr.3319 PMC7228407 32233013

[42] Huang S Wang J Liu F Liu J Cao G Yang C. COVID-19 patients with hypertension have more severe disease: a multicenter retrospective observational study. Hypertension Res. (2020) 43:824–31. doi: 10.1038/s41440-020-0485-2, PMID: 32483311 PMC7261650

[43] Corona G Giuliani C Parenti G Norello D Verbalis JG Forti G. Moderate hyponatremia is associated with increased risk of mortality: evidence from a meta-analysis. PloS One. (2013) 8:e80451. doi: 10.1371/journal.pone.0080451 24367479 PMC3867320

[44] Gheorghe G Ilie M Bungau S Stoian AMP Bacalbasa N Diaconu CC. Is there a relationship between COVID-19 and hyponatremia? Medicina (Kaunas). (2021) 57:1–7. doi: 10.3390/medicina57010055 PMC7827825 33435405

[45] Spital A. Diuretic-induced hyponatremia. Am J Nephrol. (1999) 14607:447–52. doi: 10.1159/000013496 10460932

[46] Liamis G Milionis H Elisaf M. A review of drug-induced hyponatremia. Am J Kidney Dis. (2008) 52:144–53. doi: 10.1053/j.ajkd.2008.03.004, PMID: 18468754

[47] José Carlos de la Flor M Francisco Valga A Alexander M Miguel Rodeles del P. Hyponatremia in COVID-19 infection - possible causal factors and management. JCNRC. (2020) 6:S522–4. doi: 10.23937/2572-3286.1510057

[48] Behl T Kaur I Bungau S Kumar A Uddin MS Kumar C. The dual impact of ACE2 in COVID-19 and ironical actions in geriatrics and pediatrics with possible therapeutic solutions. Life Sci. (2020) 257:118075. doi: 10.1016/j.lfs.2020.118075, PMID: 32653522 PMC7347488

[49] Sanz AB Sanchez-Nĩo MD Ortiz A. TWEAK, a multifunctional cytokine in kidney injury. Kidney Int. (2011) 80:708–18. doi: 10.1038/ki.2011.180, PMID: 21697814

[50] Werion A Belkhir L Perrot M Schmit G Aydin S Chen Z. SARS-CoV-2 causes a specific dysfunction of the kidney proximal tubule. Kidney Int. (2020) 98:1296–307. doi: 10.1016/j.kint.2020.07.019, PMID: 32791255 PMC7416689

[51] Tanaka S De Tymowski C Assadi M Zappella N Jean-Baptiste S Robert T. Lipoprotein concentrations over time in the intensive care unit COVID-19 patients: results from the ApoCOVID study. PloS one. (2020) 15:e0239573. doi: 10.1371/journal.pone.0239573, PMID: 32970772 PMC7514065

[52] Fan J Wang H Ye G Cao X Xu X Tan W. Letter to the editor: low-density lipoprotein is a potential predictor of poor prognosis in patients with coronavirus disease 2019. Metab Clin Exp. (2020) 107:154243. doi: 10.1016/j.metabol.2020.154243, PMID: 32320740 PMC7166305

[53] Hu X Chen D Wu L He G Ye W. Declined serum high density lipoprotein cholesterol is associated with the severity of COVID-19 infection. Clin Chim Acta. (2020) 510:105–10. doi: 10.1016/j.cca.2020.07.015, PMID: 32653486 PMC7350883

[54] Wang G Zhang Q Zhao X Dong H Wu C Wu F. Low high-density lipoprotein level is correlated with the severity of COVID-19 patients: an observational study. Lipids Health Dis. (2020) 19:1–7. doi: 10.1186/s12944-020-01382-9 32892746 PMC7475024

[55] Begue F Tanaka S Mouktadi Z Rondeau P Veeren B Diotel N. Altered high-density lipoprotein composition and functions during severe COVID-19. Sci Rep. (2021) 11:1–16. doi: 10.1038/s41598-021-81638-1 33504824 PMC7841145

[56] Ressaire Q Dudoignon E Moreno N Coutrot M Dépret F. Low total cholesterol blood level is correlated with pulmonary severity in COVID-19 critical ill patients. Anaesth Crit Care Pain Med. (2020) 39:733–5. doi: 10.1016/j.accpm.2020.07.015, PMID: 32866665 PMC7455102

[57] Emancipator K Csako G Elin RJ. In vitro inactivation of bacterial endotoxin by human lipoproteins and apolipoproteins. Infect Immun. (1991) 60:596–601. doi: 10.1128/iai.60.2.596-601.1992 PMC257670 1730494

[58] Munford RS. Detoxifying endotoxin: time, place and person. J Endotoxin Res. (2005) 11:69–84. doi: 10.1179/096805105X35161, PMID: 15949133

[59] Lee R-P Lin N-T Chao Y-FC Lin C-C Harn H-J Chen H-I. High-density lipoprotein prevents organ damage in endotoxemia. Res Nurs Health. (2007) 30:250–60. doi: 10.1002/nur.20187, PMID: 17514720

[60] Grunfeld C Marshall M Shigenaga JK Moser AH Tobias P Feingold KR. Lipoproteins inhibit macrophage activation by lipoteichoic acid. J Lipid Res. (1999) 40:245–52. doi: 10.1016/S0022-2275(20)33363-0, PMID: 9925653

[61] Feingold KR Grunfeld C. Lipids: a key player in the battle between the host and microorganisms. J Lipid Res. (2012) 53:2487–9. doi: 10.1194/jlr.E033407, PMID: 23075464 PMC3494250

[62] Petrilli CM Jones SA Yang J Rajagopalan H O’Donnell L Chernyak Y. Factors associated with hospital admission and critical illness among 5279 people with coronavirus disease 2019 in new York City: prospective cohort study. BMJ. (2020) 369:m1966. doi: 10.1136/bmj.m1966 32444366 PMC7243801

[63] Banach M Penson PE Fras Z Vrablik M Pella D Reiner Ž. Brief recommendations on the management of adult patients with familial hypercholesterolemia during the COVID-19 pandemic. Pharmacol Res. (2020) 158:104891. doi: 10.1016/j.phrs.2020.104891, PMID: 32389859 PMC7204727

[64] Iqbal Z Ho JH Adam S France M Syed A Neely D. Managing hyperlipidaemia in patients with COVID-19 and during its pandemic: an expert panel position statement from HEART UK. Atherosclerosis. (2020) 313:126–36. doi: 10.1016/j.atherosclerosis.2020.09.008, PMID: 33045618 PMC7490256

[65] Vuorio A Raal F Kaste M Kovanen PT. Familial hypercholesterolaemia and COVID-19: a two-hit scenario for endothelial dysfunction amenable to treatment. Atherosclerosis. (2021) 320:53–60. doi: 10.1016/j.atherosclerosis.2021.01.021, PMID: 33540179 PMC7830285

[66] Sorensen KE Celermajer DS Georgakopoulos D Hatcher G Betteridge DJ Deanfield JE. Impairment of endothelium-dependent dilation is an early event in children with familial hypercholesterolemia and is related to the lipoprotein(a) level. J Clin Invest. (1994) 93:50–5. doi: 10.1172/JCI116983, PMID: 8282821 PMC293724

[67] Charakida M Tousoulis D Skoumas I Pitsavos C Vasiliadou C Stefanadi E. Inflammatory and thrombotic processes are associated with vascular dysfunction in children with familial hypercholesterolemia. Atherosclerosis. (2009) 204:532–7. doi: 10.1016/j.atherosclerosis.2008.09.025, PMID: 19004443

[68] Haitao T Vermunt JV Abeykoon J Ghamrawi R Gunaratne M Jayachandran M. COVID-19 and sex differences. Mayo Clin Proc. (2020) 95:2189–203. doi: 10.1016/j.mayocp.2020.07.024, PMID: 33012349 PMC7402208

[69] Zhang C Shi L Wang FS. Liver injury in COVID-19: management and challenges. Lancet Gastroenterol Hepatol. (2020) 5:428–30. doi: 10.1016/S2468-1253(20)30057-1, PMID: 32145190 PMC7129165

[70] Ronco C Reis T Husain-Syed F. Management of acute kidney injury in patients with COVID-19. Lancet Respir Med. (2020) 8:738–42. doi: 10.1016/S2213-2600(20)30229-0, PMID: 32416769 PMC7255232

[71] Han H Ma Q Li C Liu R Zhao L Wang W. Profiling serum cytokines in COVID-19 patients reveals IL-6 and IL-10 are disease severity predictors. Emerg Microbes Infect. (2020) 9:1123–30. doi: 10.1080/22221751.2020.1770129, PMID: 32475230 PMC7473317

[72] Kaiser R Leunig A Pekayvaz K Popp O Joppich M Polewka V. Self-sustaining IL-8 loops drive a prothrombotic neutrophil phenotype in severe COVID-19. JCI Insight. (2021) 6:1–15. doi: 10.1172/jci.insight.150862 PMC8492337 34403366

[73] Wynants L Van Calster B Collins GS Riley RD Heinze G Schuit E. Prediction models for diagnosis and prognosis of covid-19: systematic review and critical appraisal. BMJ. (2020) 369:m1328. doi: 10.1136/bmj.m1328, PMID: 32265220 PMC7222643

[74] Tahir Huyut M Huyut Z İlkbahar F Mertoğlu C. What is the impact and efficacy of routine immunological, biochemical and hematological biomarkers as predictors of COVID-19 mortality? Int Immunopharmacol. (2022) 105:108542. doi: 10.1016/j.intimp.2022.108542, PMID: 35063753 PMC8761578

[75] Huyut MT İlkbahar F. The effectiveness of blood routine parameters and some biomarkers as a potential diagnostic tool in the diagnosis and prognosis of Covid-19 disease. Int Immunopharmacol. (2021) 98:107838. doi: 10.1016/j.intimp.2021.107838, PMID: 34303274 PMC8169318

