# Supplementary material for: Retrospective cohort study to evaluate the continuous use of anticholesterolemics and diuretics in patients with COVID-19
Source: Front Med (Lausanne). 2024 Jan 11;10:1252556. doi: 10.3389/fmed.2023.1252556 (PMC10808793; doi:10.3389/fmed.2023.1252556)
Supplement: Supplementary file 1 [file Table_1.DOCX]

**Supplementary file 1**

**Clinical characteristics**


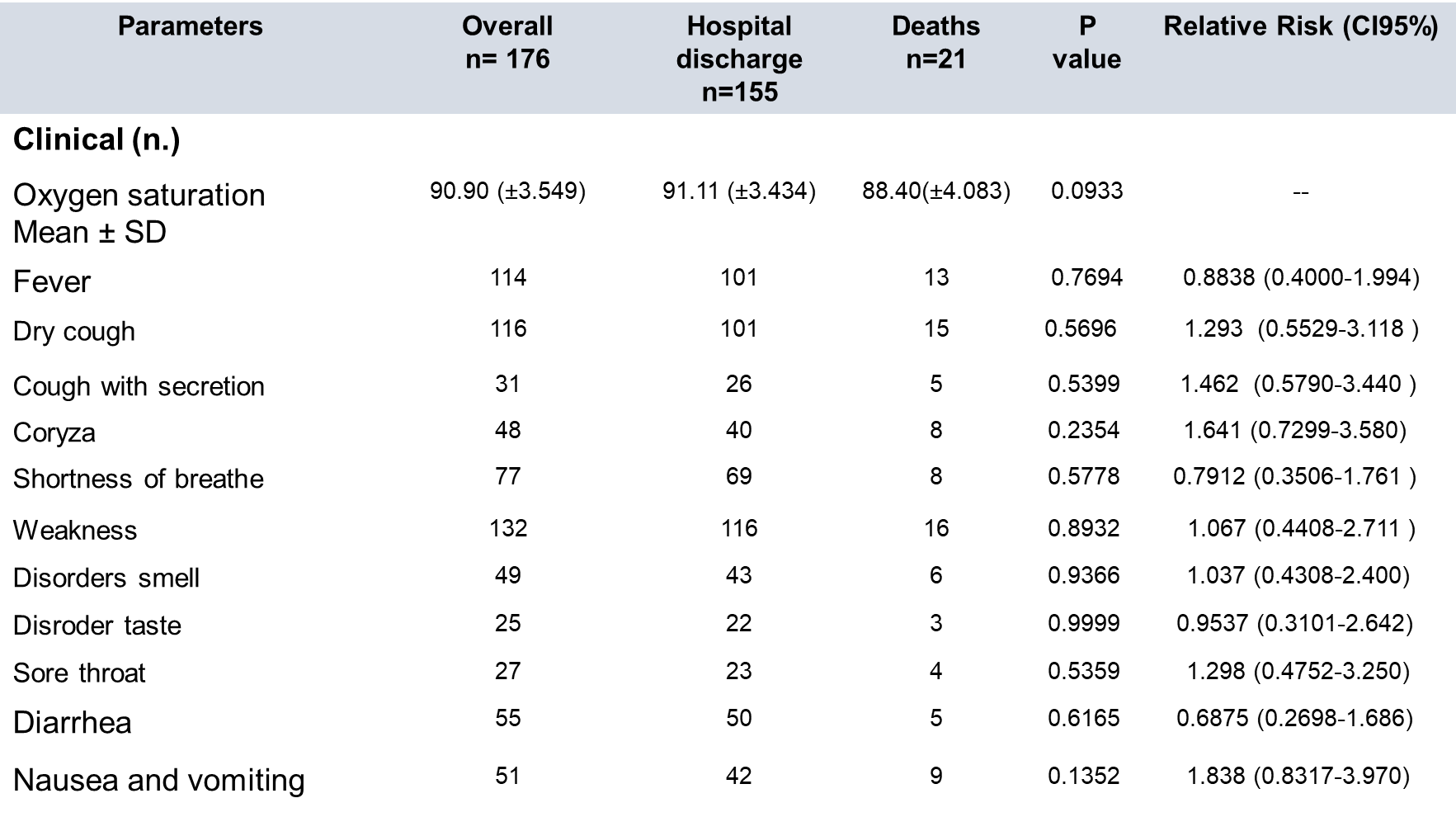


Data are presented as mean ± SD or median and interquartile range (Minimum- Maximum). CI = confidence interval
