# Supplementary material for: Retrospective cohort study to evaluate the continuous use of anticholesterolemics and diuretics in patients with COVID-19
Source: Front Med (Lausanne). 2024 Jan 11;10:1252556. doi: 10.3389/fmed.2023.1252556 (PMC10808793; doi:10.3389/fmed.2023.1252556)
Supplement: Supplementary file 2 [file Table_2.DOCX]

**Laboratory test results**


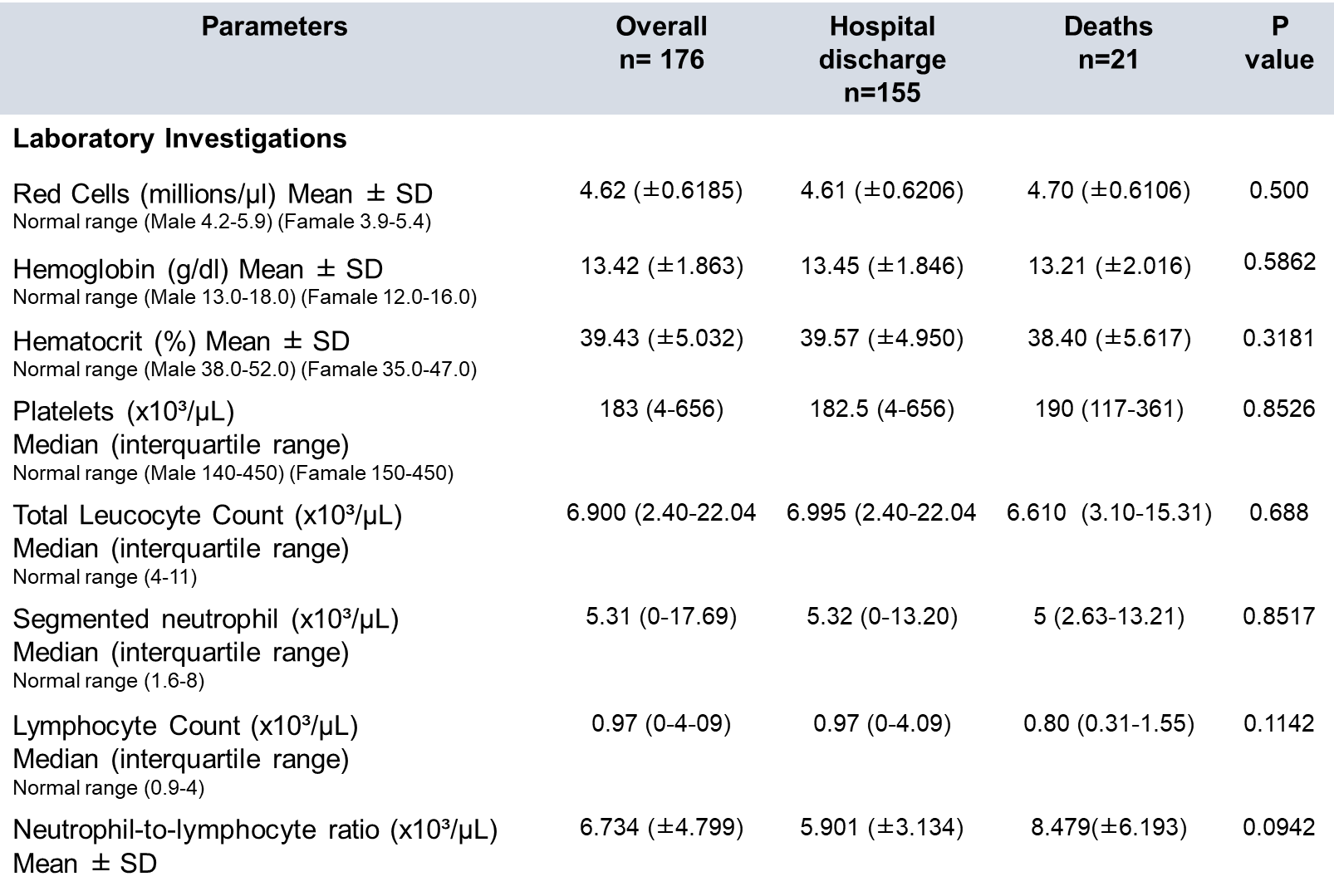


Data are presented as mean ± standard deviation (SD) or median and (Minimum-Maximum interquartile range) or were presented as Relative Risk and Confidence Intervals (CI) 95%.
