# Supplementary figures and images for: Retrospective cohort study to evaluate the continuous use of anticholesterolemics and diuretics in patients with COVID-19
Source: Front Med (Lausanne). 2024 Jan 11;10:1252556. doi: 10.3389/fmed.2023.1252556 (PMC10808793; doi:10.3389/fmed.2023.1252556)

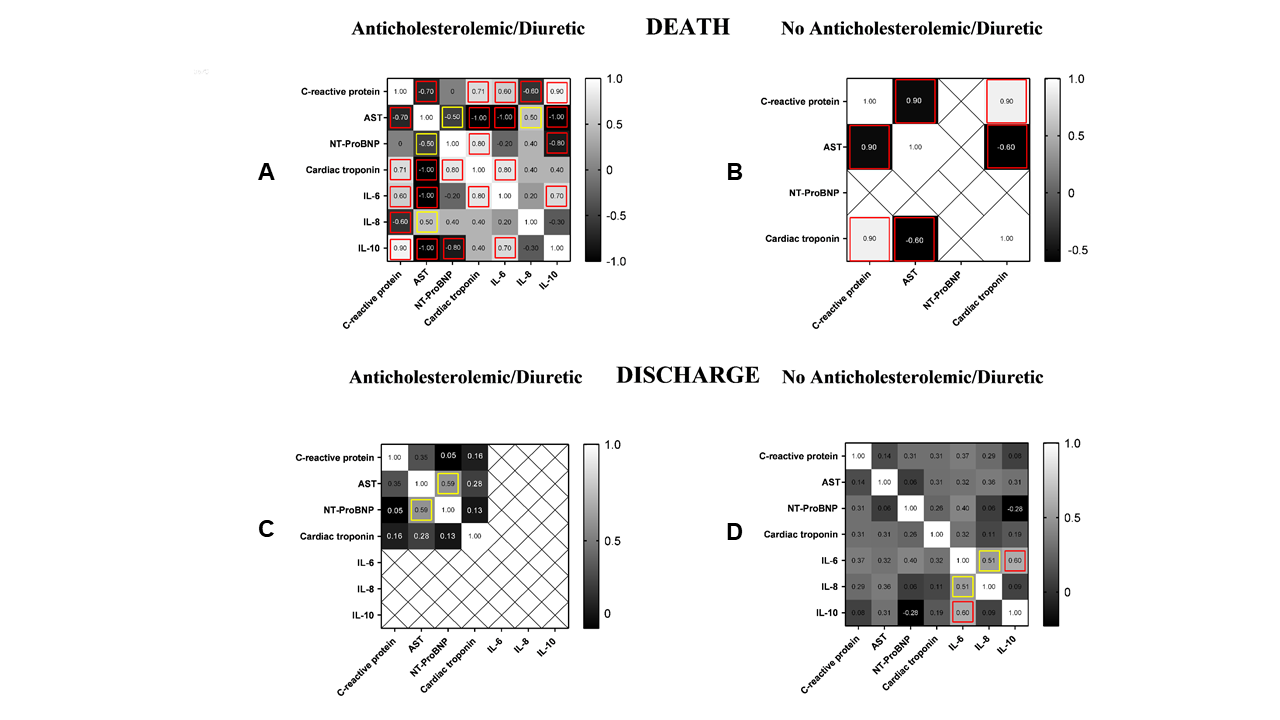

Supplement: Supplementary file 3 [file Image_1.TIF]
